# MITF in melanoma: mechanisms behind its expression and activity

**DOI:** 10.1007/s00018-014-1791-0

**Published:** 2014-11-30

**Authors:** Mariusz L. Hartman, Malgorzata Czyz

**Affiliations:** Department of Molecular Biology of Cancer, Medical University of Lodz, 6/8 Mazowiecka Street, 92-215 Lodz, Poland

**Keywords:** BRAF^V600E^, Melanoma cancer plasticity, Gene regulation, MicroRNA, MITF, Oncogene, Transcription factor

## Abstract

MITF (microphthalmia-associated transcription factor) represents a melanocytic lineage-specific transcription factor whose role is profoundly extended in malignant melanoma. Over the last few years, the function of MITF has been tightly connected to plasticity of melanoma cells. MITF participates in executing diverse melanoma phenotypes defined by distinct gene expression profiles. Mutation-dependent alterations in MITF expression and activity have been found in a relatively small subset of melanomas. MITF activity is rather modulated by its upstream activators and suppressors operating on transcriptional, post-transcriptional and post-translational levels. These regulatory mechanisms also include epigenetic and microenvironmental signals. Several transcription factors and signaling pathways involved in the regulation of MITF expression and/or activity such as the Wnt/β-catenin pathway are broadly utilized by various types of tumors, whereas others, e.g., BRAF^V600E^/ERK1/2 are more specific for melanoma. Furthermore, the MITF activity can be affected by the availability of transcriptional co-partners that are often redirected by MITF from their own canonical signaling pathways. In this review, we discuss the complexity of a multilevel regulation of MITF expression and activity that underlies distinct context-related phenotypes of melanoma and might explain diverse responses of melanoma patients to currently used therapeutics.

## Introduction

Melanocytes are pigment-producing cells whose differentiation, proliferation and survival largely depend on MITF (microphthalmia-associated transcription factor), a melanocyte-specific transcription factor [for review 1, 2]. Melanoma is a melanocyte-derived tumor in which MITF dependence is retained [for review 3]; thus, MITF represents a lineage-restricted regulator that operates in normal cells, and its activity is also used by malignant cells. Enforced expression of MITF in immortalized melanocytes [4] or neural crest progenitor cells [5] when introduced together with melanoma-specific BRAF^V600E^ suggests MITF’s role as a melanoma addiction oncogene. MITF is recognized as a driver of melanoma progression [for review 6], but its role in suppression of invasion and metastasis has been also shown [7–10]. By activating the expression of almost one hundred genes, MITF can regulate multiple biological processes in melanoma cells such as differentiation, proliferation, migration, and senescence [11–13; for review 14, 15]. MITF also exerts pro-survival role by activating the expression of anti-apoptotic genes including *BCL2A1*, *BCL2* and *BIRC7* (ML-IAP/livin) [for review 16, 17]. Recent studies implicate MITF in energy metabolism and organelle biogenesis [18; for review 19]. This variety of often mutually exclusive cellular programs driven by MITF stands for distinct phenotypes of melanoma cells [12, 20, 21; for review 22, 23]. MITF is also recognized as a major regulator in a “phenotypic switching” concept explaining a high plasticity of melanoma cells [20, 21, 24–27; for review 22, 28]. Therefore, better understanding of the intracellular mechanisms underlying a contextual regulation of MITF is of utmost importance. In this review, we focus on melanoma-related mechanisms underlying the regulation of MITF expression and activity.

## Gene structure and transcriptional regulation of *MITF*

In human, *MITF* locus is mapped to chromosome 3 and spans 229 kbp. *MITF* encodes a b-HLH-Zip (basic helix-loop-helix leucine zipper) transcription factor that belongs to the MYC superfamily. Together with TFEB, TFEC and TFE3, MITF constitutes the MiT (microphthalmia) family of transcription factors [29]. All of them share a common b-HLH-Zip dimerization motif containing a positively charged fragment involved in DNA binding, and a transactivation domain (TAD) [29]. As a result of differential usage of alternative promoters, a single *MITF* gene produces several isoforms including MITF-A [30], MITF-B [31], MITF-C [32], MITF-D [33], MITF-E [34], MITF-H [35], MITF-J [36], MITF-Mc [37] and MITF-M [38, 39]. These isoforms differ in their N-termini encoded by exon 1, and show tissue-specific pattern of expression. The expression of the shortest isoform MITF-M (a 419-residue protein) is limited to melanocytes and melanoma cells [39; for review 40]. MITF-Mdel, a variant of MITF-M harboring two in-frame deletions within the exons 2 and 6, has been identified as restrictedly expressed in these cells [41]. MITF contains two TADs responsible for its transcriptional activity; however, a functional domination of the TAD at N-terminus over that one at C-terminus has been reported [42]. MITF binds to DNA as a homodimer or heterodimer with one of the MiT proteins [29], but does not form heterodimers with other b-HLH-Zip transcription factors such as MYC, MAX and USF, despite a common ability to bind to the palindromic CACGTG E-box motif [43]. It was shown that the heptad repeat register of the leucine zipper in MITF is broken by a three-residue insertion that generates a kink in one of the two zipper helices, which limits the ability of MITF to form dimers only with those bHLHZip transcription factors that contain the same type of insertion [43]. Functionally, the MITF-binding sites in the promoters of target genes involve E-box: CA[C/T]GTG and M-box, extended E-box with an additional 5′-end flanking thymidine nucleotide: TCATGTGCT [for review 44].

### Genetic alterations in *MITF* and alternative splicing

Some genetic alterations have been associated with *MITF*. Initially, high-density single nucleotide polymorphism arrays revealed the *MITF* amplification in up to 20 % of melanomas, with higher incidence among metastatic melanoma samples [4]. This aberration correlated with decreased overall patient survival [4]. However, in a recent study involving targeted-capture deep sequencing, no copy gains at the *MITF* locus have been found in a panel of melanoma metastases [45]. Genetic abnormalities related to *MITF* also include single base substitutions in the regions encoding its functional domains [46]. These somatic mutations, however, do not affect the DNA-binding ability of MITF in melanoma cells [47]. Recently, two independent studies have identified a rare oncogenic MITF^E318K^ variant representing a gain-of-function allele for MITF that is present in patients with familial melanoma and a small fraction of sporadic melanomas [48, 49]. *MITF* E318K has been described as a medium-penetrance gene in melanoma associated with multiple primary melanomas developed in its carriers [50, 51], and as predisposing to renal carcinoma as well [48].

Alternative splicing is another mechanism of MITF regulation in melanoma. Two spliced variants of MITF, MITF(+) containing an internal six-amino acid fragment encoded by exon 6a and MITF(−) that lacks this fragment, have been described. These two variants possess different activity, with anti-proliferative property of MITF(+). This effect is tightly related to the interaction between the N-terminal fragment of MITF(+) with its specific hexapeptide [52]. Activation of the MEK1-ERK2 (extracellular signal-regulated kinase 2) pathway, independently of the mutational status of *BRAF* and *NRAS*, has been indicated as a mechanism underlying the expression of MITF splice variants [53]. Additionally, the quantification of these variants in a panel of 86 melanoma samples revealed the apparently increased expression of MITF(−) in metastatic melanomas [53].

### Transcriptional activators of MITF

**Fig. 1:**
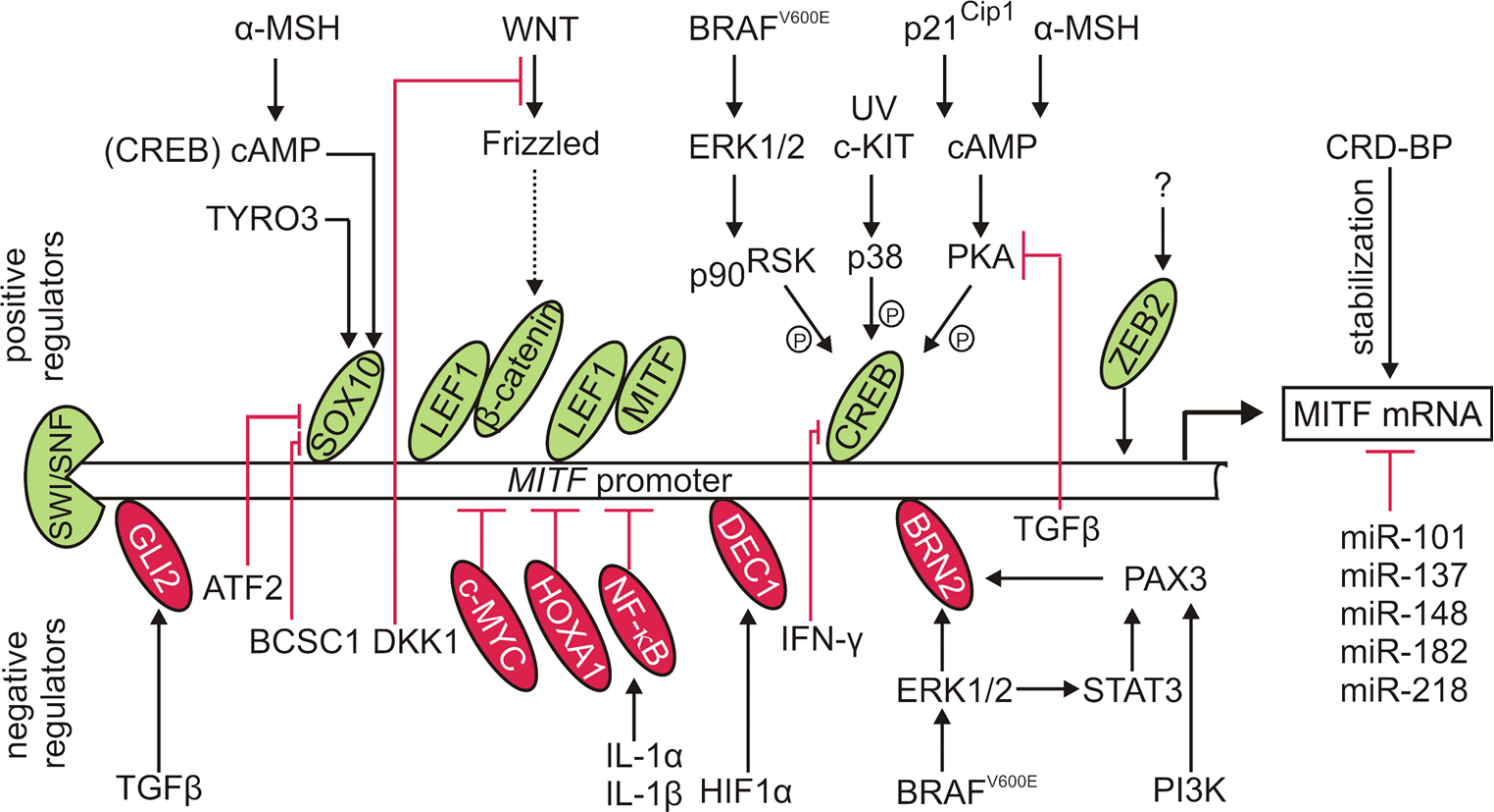
Transcriptional and post-transcriptional regulation of MITF expression. The variety of MITF regulators, activated by diverse signaling pathways often modified in melanoma, comprises a number of transcription factors either promoting MITF expression (positive regulators; shown in *green*) or inhibiting its transcription (negative regulators; shown in *red*). Upstream regulators of these transcription factors may indirectly affect MITF level. A correlation is also observed between MITF level and activity of transcription factors, e.g., NF-κB, not operating on MITF promoter. Moreover, a chromatin-remodeling complex SWI/SNF promotes MITF expression. In addition, MITF transcript can be either negatively regulated by miRs, or stabilized by the association with CRD-BP

The transcriptional control of *MITF* is governed by a number of transcription factors and their regulators associated with signaling pathways involved in diverse cellular processes (Fig. 1). SOX10 (sex-determining region Y-box 10)-responsive element was found between −264 and −266 in the *MITF* promoter [54]. In addition, an activating frameshift or non-sense mutations in *SOX10* have been identified in melanoma cells, and *MITF* and *SOX10* have been found mutated in a mutually exclusive manner [46]. The nuclear localization of SOX10 is maintained by a protein tyrosine kinase TYRO3 [55]. SOX10 also cooperates with CREB (cAMP response element-binding protein) in the responsiveness of *MITF* to α-MSH (α-melanocyte-stimulating hormone)-cAMP signaling. This constitutes a tightly restricted mechanism of regulation due to a ubiquitous expression of CREB and a cell type-limited expression of SOX10 [56]. CREB is targeted by a number of regulators that promote its phosphorylation at Ser^133^, thus activating CREB-dependent transcription (Fig. 1) [57; for review 58]. It has been demonstrated that p38, activated by either UV (ultraviolet radiation) or receptors, e.g., KIT, phosphorylates CREB and promotes its binding to the *MITF* promoter [57]. p21^Cip1^, a cell cycle inhibitor, has been identified as a CREB co-factor involved in cAMP-dependent MITF expression in melanoma [59]. MITF expression can be also mediated by the complex of two key effectors of the Wnt (wingless-type) signaling pathway, LEF1 (lymphoid enhancer-binding factor 1) and β-catenin [20, 60]. In contrast to β-catenin, a phenotype-specific expression of LEF1 has been shown in melanoma cells limiting LEF1/β-catenin-dependent *MITF* transcription to a defined cellular context [20]. Importantly, MITF can cooperate with LEF1 as a non-DNA-binding coactivator to enhance its own expression [60]. It has been also demonstrated that mediators of α-MSH/cAMP/PKA (protein kinase A) signaling can redirect β-catenin to the CREB-specific promoters to activate transcription of CREB target genes including *MITF* [61]. Most recently, a transcription factor involved in epithelial–mesenchymal transition [62; for review 63, 64], ZEB2 (zing finger E-box binding protein 2) has been shown to activate MITF expression, and a ZEB2 loss that resulted in a decreased MITF level and several MITF-dependent target genes was associated with melanoma progression [65]. In contrast to activating potential of ZEB2 on the *MITF* promoter, ZEB1 has been found to directly repress MITF expression in retinal pigment epithelium [66]. Thus, the role of ZEB1 in the context of MITF expression in melanoma needs to be elucidated. On the level of chromatin remodeling, it has been demonstrated that a SWI/SNF complex containing BRM (Brahma) or BRG1 (Brahma-related gene 1) promotes MITF expression [67].

### Transcriptional repressors of MITF

Several transcription factors have been identified as direct repressors of MITF (Fig. 1). Inverse expression of GLI2 (glioma-associated oncogene family member 2) and MITF-M, relating to the mutually exclusive transcriptional programs, has been observed in melanoma cells [26]. GLI2 is a Kruppel-like transcription factor activated by TGFβ (transforming growth factor β) [26]. Furthermore, a GLI2 binding site was identified in the −334/−296 region of the *MITF* promoter confirming direct inhibitory activity of GLI2 towards *MITF* [68]. The contribution of PAX3 (paired box 3) to MITF expression represents another melanoma-specific mechanism. Although positive regulation of MITF by PAX3 in melanocytes is well described [69], PAX3 is thought to function independently of MITF [70] or even play a repressive role on MITF expression in melanoma [21]. PAX3 is activated by PI3 K (phosphatidylinositol 3-kinase) [71] and STAT3 (signal transducer and activator of transcription 3) [72] in melanoma cells, and a negative PAX3-dependent regulation of *MITF* expression is mediated by BRN2 encoded by *POU3F2* [21, 71]. Notably, BRN2-mediated repression of *MITF* transcription represents a mechanism distinguishing between melanoma cells and melanocytes due to the lack of BRN2 expression in the latter, which might be explained by the involvement of melanoma-specific BRAF^V600E^ in BRN2 upregulation [73]. Moreover, inverse expression of MITF and BRN2 was shown in vitro [8] and in vivo [7]. The antagonistic MITF and PAX3 expression has been proposed as a switch model in which MITF and miR-211, residing in the sixth intron of *TRPM1*, can activate one cellular program while suppressing another one driven by PAX3 and BRN2 [9, 21, 74]. MITF repression is also mediated by DEC1 (differentially expressed in chondrocytes protein 1) whose recruitment to the *MITF* promoter is regulated by HIF1 (hypoxia-inducible factor 1) [75]. As HIF1α is a MITF target [76] and can be expressed in melanoma cells not only under hypoxic conditions [77], this mechanism constitutes an interesting negative feedback loop regulating MITF expression.

Several proteins have been found to indirectly suppress MITF expression by acting as upstream inhibitors of positive regulators of MITF expression (Fig. 1). Independently of its effect on GLI2, TGFβ inhibits PKA that otherwise promotes CREB-dependent *MITF* transcription [68]. DKK1 (Dickkopf-1), a secreted inhibitor of the Wnt/β-catenin pathway, has been shown to suppress both MITF expression and the MITF-dependent differentiation program [78]. Accordingly, both DKK1 expression and secretion have been substantially reduced in the multicellular anchorage-independent melanospheres showing high expression of MITF and numerous MITF target genes [79]. ATF2 (activating transcription factor 2) displays inhibitory activity towards SOX10 both in melanocytes and melanoma cells, resulting in a decreased MITF transcript level [80]. A co-immunoprecipitation approach confirmed selective affinity of BCSC1 (breast cancer suppressor candidate-1) to SOX10, but not other *MITF* regulators such as CREB, and down-regulated MITF mRNA level was observed upon BCSC-1 overexpression [81]. MITF expression is also affected by IFN-γ (interferon γ) that inhibits CREB binding to the *MITF* promoter by inducing the association of CBP (CREB binding protein) with STAT1 [82].

## Regulation of MITF transcript stability

Melanoma cells show high expression of CRD-BP (coding region determinant-binding protein), an mRNA-binding protein that has been found to stabilize MITF transcript [83]. MITF transcript is also under control of several small non-coding RNAs, microRNAs (miRs), which promote mRNA degradation or suppress protein synthesis via binding to 3′-UTR of a target transcript [23]. miR-137, located in the locus 1p22, negatively regulates MITF [84, 85]. No mutations have been found in the putative miR-137-binding sites in the MITF mRNA 3′-UTR, however, miR-137 possesses a 15-bp tandem repeat in the pre-miR-137 sequence that alters the processing and function of miR-137 in melanoma cell lines [84]. In metastatic melanoma samples, MITF transcript has been determined as a target of miR-182-mediated degradation [86]. miR-182 is a member of the miR cluster residing in a chromosomal locus (7q31–34) frequently amplified in melanomas. Interestingly, overexpression of MITF has been related to the suppression of the miR-182-dependent pro-invasive effect [86]. p53-dependent miR-182 has been also found to down-regulate MITF in uveal melanoma [87]. The 3′-UTR of MITF transcript is also targeted by miR-148 [88], miR-101 [89] and miR-218, and inverse correlation between MITF and miR-218 has been observed in melanocytes and melanoma cell lines [90]. Notably, exosome-dependent miR exchange between melanoma cells may influence MITF transcript level as well [for review 91].

## Regulation of MITF protein level and activity

**Fig. 2:**
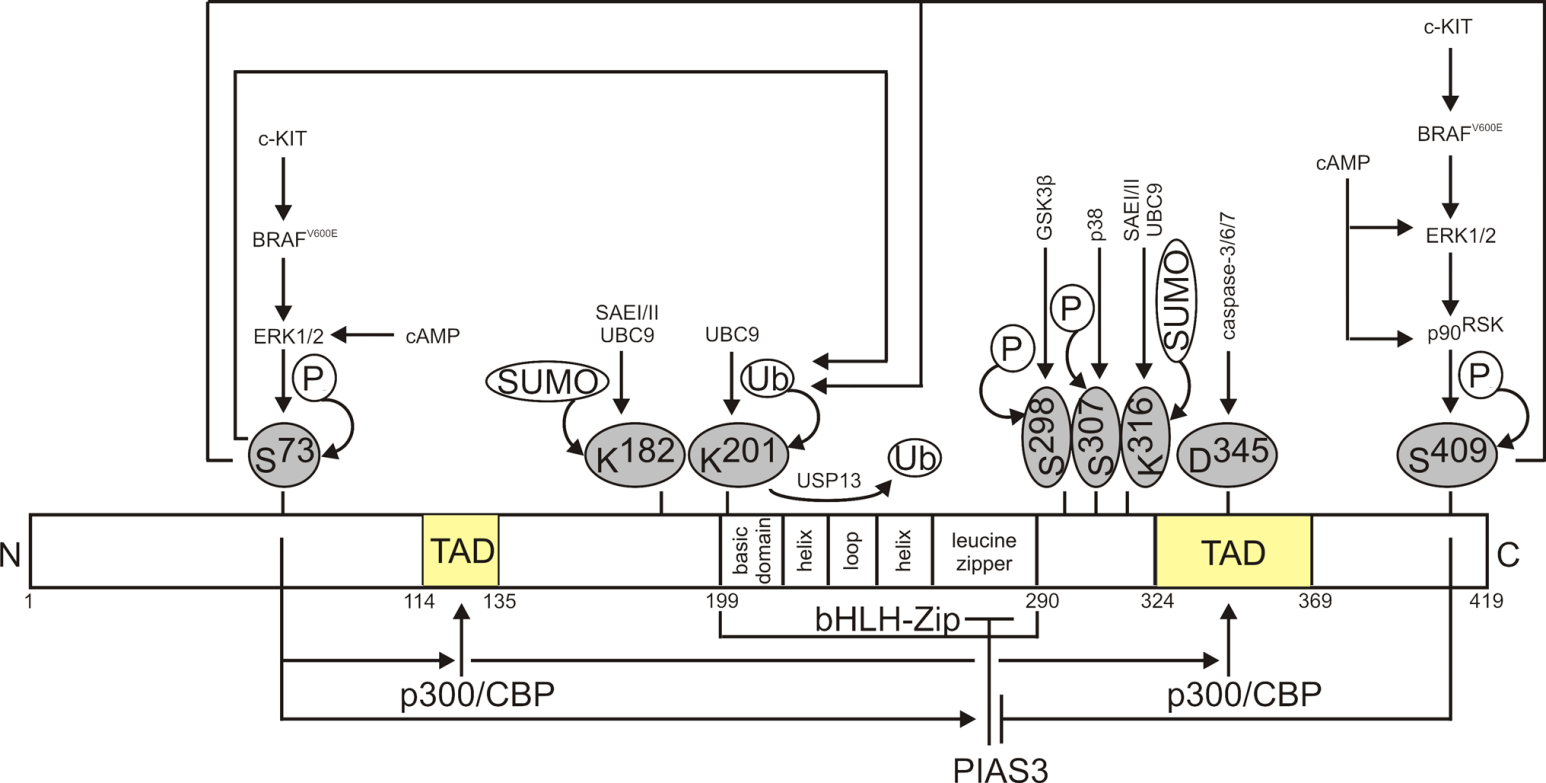
A schematic domain structure of MITF-M protein, a melanocyte/melanoma-specific isoform, and its key regulatory mechanisms. MITF-M comprises 419 amino acids. The functional domains of MITF-M common for all isoforms are encoded by the exons from 2 to 9. Phosphorylation enhances the transcriptional activity of MITF. However, this modification may also promote proteasome-dependent degradation of MITF, or enhance interaction between MITF and p300/CBP. MITF can be subjected to USP13-mediated deubiquitylation, thus preventing MITF from proteasomal degradation. In addition, MITF can be a target for other modifications, including SUMOylation and caspase-mediated cleavage

The transcriptional activity of MITF depends on its post-translational modifications and availability of co-operating partners (Fig. 2). MITF can be regulated by phosphorylation maintained by ERK1/2 (at Ser^73^), p90^RSK^ (at Ser^409^) [92], GSK3β (at Ser^298^) [93, 94] and p38 (at Ser^307^) [95]. In general, phosphorylation enhances transcriptional activity of MITF [94, 95; for review 96]. The phosphorylation at Ser^73^ promotes the interaction with a MITF co-factor, histone acetyl transferase p300/CBP within the transactivation domain of MITF [92]. On the other hand, this modification promotes the binding of PIAS3 (protein inhibitor of activated STAT3) that involves the N-terminal fragment of PIAS3 and the leucine zipper of MITF [97, 98]. Interaction with PIAS3 leads to the attenuation of MITF transcriptional activity. This effect is, however, inhibited when MITF is phosphorylated at Ser^409^ in a p90^RSK^-dependent manner [97]. The phosphorylation of MITF at Ser^73^, a residue located within a degradation-promoting PEST sequence, is also a prerequisite to the MITF proteasome-dependent turnover [99] e.g., in response to ultraviolet C radiation [100]. Lys^201^ has been identified as a site of UBC9-mediated ubiquitylation of MITF [99]. Proteasome-mediated MITF protein degradation has also been observed after double phosphorylation at Ser^73^ and Ser^409^ [92]. An unphosphorylatable mutant at Ser^73^/Ser^409^ has been very stable but transcriptionally incompetent [92], indicating that signals promoting transcriptional activity and degradation of MITF protein are coupled in melanoma cells. Both phosphorylations promoting MITF degradation depend on melanoma-specific BRAF^V600E^ causing the enhanced activation of MAPK (MAP kinase)/ERK1/2 pathway [101]. Deubiquitinase USP13 (ubiquitin-specific protease 13) has been linked to the protection of MITF from proteasomal degradation in melanoma cells [102]. MITF can be also processed by the effector caspases. It has been demonstrated in melanoma cells that MITF-derived C-terminal peptide cleaved by these proteases has a pro-apoptotic function [103].

MITF activity is also modulated by SUMOylation at two lysine residues, Lys^182^ and Lys^316^ [104]. SUMOylation of MITF depends on an E1 SUMO-activating heterodimeric enzyme SAEI/SAEII and E2 SUMO-conjugating enzyme UBC9 [104] (Fig. 2). It has been concluded that this modification plays an essential role in the regulation of MITF activity, and non-SUMOylatable MITF mutants displayed increased transcriptional activity on distinct sets of target genes [48, 49, 104, 105]. It has been also indicated that PIAS3 can promote MITF SUMOylation at both SUMOylation sites [105]; however, this observation has not been clearly supported by another report [104]. A recent study on melanoma patients bearing the MITF^E318K^ variant [51], in which a point mutation occurs at the consensus binding site for SUMOylation [105], supports the conclusion that this substitution does not affect MITF protein stability and nuclear localization.

Besides aforementioned regulations, MITF activity also depends on the availability of co-operating partners such as p300/CBP [92]. p300/CBP is a versatile regulator that links transcription factors bound to DNA with a basal transcriptional machinery, thus promoting the assembly of pre-initiation complex [for review 106]. Immunofluorescence and immunoprecipitation studies have demonstrated that MITF can physically interact with BRM and BRG1, and depending on the type of SWI/SNF complex composed of either the BRG1 or BRM subunit, different sets of MITF-dependent genes are activated [107]. This, however, is not an exclusive role of the BRG1-containing SWI/SNF complex in melanoma since its MITF-independent activity has been shown as well [108]. SOX10 can synergistically activate the MITF-dependent genes as demonstrated for *MET* [109]. It has been also shown that MITF can redirect β-catenin from the Wnt signaling pathway, and engage it to the activation of MITF-dependent genes [110]. Thus, SWI/SNF complex, SOX10 and β-catenin can function not only as activators of MITF expression (Fig. 1), but also as its co-factors. HINT1 (histidine triad nucleotide-binding protein 1) has been identified as an inhibitor of transcriptional activity of MITF acting through binding the chromatin at the MITF sites [111]. Moreover, since HINT1 expression is lost in primary melanomas [111], it may support a role of MITF in melanomagenesis.

The mechanisms behind the regulation of MITF level and activity are still being explored. Most recently, very interesting correlations have been observed between activity of MITF and other transcription factors, probably not operating on the *MITF* promoter (Fig. 1). HOXA1 (homeobox transcription factor 1) has been identified as a potent inhibitor of MITF expression whose activity might be concomitant with the activation of the TGFβ pathway [112]. An interesting repression mechanism has been reported in melanoma cells expressing IL-1R (interleukin-1 receptor). The stimulation of melanoma cells with interleukin-1α or 1β resulted in the reduction of MITF expression, and it has been suggested that this process is NF-κB-dependent [113]. Accordingly, a suppressive role of the NF-κB signaling on MITF level has been reported [114]. A reverse correlation has been observed between *MYC*-related chromosomal copy number gains in 8q24 and MITF expression [115]. In contrast, suppression of ETV1 (E twenty-six variant 1) was associated with a decreased level of MITF protein [116]. Whether c-MYC and ETV1 can act as direct regulators of MITF remains to be elucidated. Other poorly characterized regulators have been associated with MITF level, and the detailed mechanisms of their actions need to be clarified. PRMT5 (protein arginine methyltransferase 5) is an enzyme involved in post-translational protein modifications. PRMT5 expression has been found to be increased in melanoma, and siRNA-mediated depletion of PRMT5 resulted in a substantial decrease in the level of MITF protein indicating a positive regulatory effect [117]. A similar influence on the MITF level has been demonstrated for PDE4D (phosphodiesterase subtype 4D), and PDE4D-depleted cells have shown a decreased MITF transcript level [118].

## Final conclusions

**Fig. 3:**
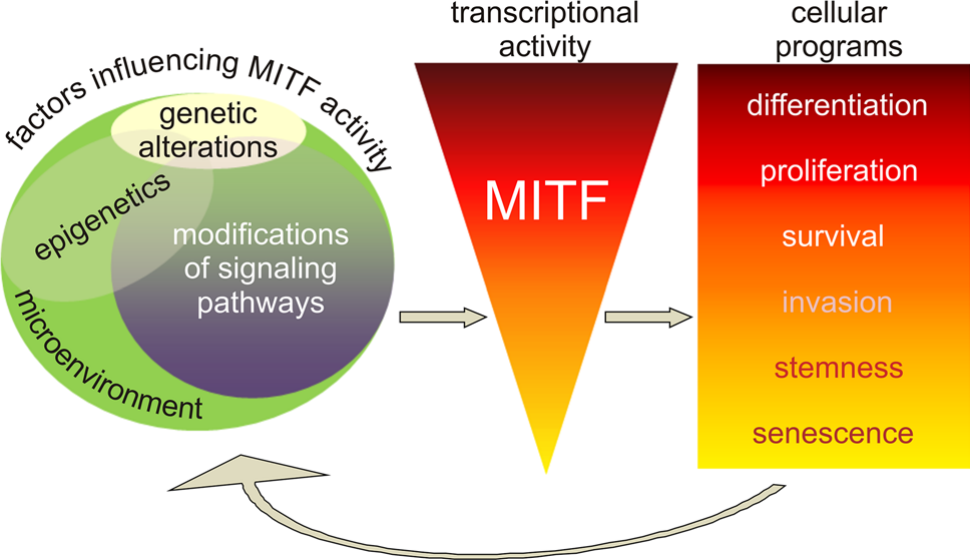
MITF expression and activity in melanoma cells are determined by genetic alterations, epigenetics, changes in upstream signaling pathways and microenvironment. Different combinations of those factors result in varied transcriptional activity of MITF which contributes to diverse cellular programs from differentiation and proliferation at high level of MITF activity to stemness and senescence at the lowest one. However, the outcome is not stable and can be modified by fluctuations in microenvironment-dependent critical epigenetic states and signaling pathways. Several MITF-dependent feedback mechanisms are also determined

MITF operates within a wide range of activity levels determining melanoma cell fate (Fig. 3) [20, 24–27; for review 22, 23, 28]. Melanoma cells expressing MITF at high level can either differentiate or proliferate. Low activity of MITF is related to stem cell-like or invasive potential. Finally, long-term MITF suppression drives cell senescence. Although genetic alterations, including mutations and amplification of *MITF*, are found in melanoma samples [4, 46, 48, 49], fluctuating MITF activity in melanoma cells is rather due to microenvironmental cues, critical epigenetic states and modifications of upstream signaling pathways [7, 8, 10, 79, 104–138]. Different combinations of those factors determine transcriptional activity of MITF which in turn contributes to diverse cellular capabilities. This may explain a variable MITF expression across melanoma specimens but also between different areas of individual tumor samples reflecting both inter-tumoral heterogeneity and diversity of melanoma cell subpopulations comprising a tumor mass [123, 124; for review 125].

MITF does not exhibit a druggable target, and MITF-aimed approaches are thought to be rather based on the modulation of its upstream regulatory pathways [126, 127]. A number of natural and synthetic compounds have been used to modify MITF activity. A few natural compounds that either reduced or increased MITF transcript level in heterogeneous patient-derived melanoma populations were identified in our laboratory [128]. A dietary flavonoid fisetin that targets Wnt/β-catenin pathway has substantially reduced MITF expression and influenced MITF-dependent cellular processes [129]. Downregulation of MITF at the transcriptional level was observed for ciglitazone that also showed anti-melanoma effects in vivo [130]. Hirsein A reduced the expression of MITF by modulating the expression of diverse components of MAPK signaling pathway [131]. Several studies have linked a high MITF level with the resistance to MAPK-pathway inhibitors [132, 133; for review 134–136]. MITF targets are up-regulated by MAPK-pathway inhibitors [137], and enforced expression of MITF in BRAF^V600E^ melanoma cells promotes resistance towards inhibitors of RAF, MEK and ERK [for review 134]. As MITF expression can be reduced by histone deacetylase inhibitors (HDACi) [138], combined HDAC and MAPK inhibition was shown to prevent MITF-driven resistance in melanoma cells [132]. Another study suggests, however, that intrinsically resistant melanomas can be characterized by low expression/activity of MITF accompanied by enhanced activity of NF-κB signaling, and BRAF inhibition in MITF-high, drug-sensitive cells induces a transition to the MITF-low/NF-κB–high state [114]. Most recent findings that modulation of MITF activity can drive phenotype switching in vivo, and abrogating MITF activity in melanoma leads to tumor regression, but a low level of wild-type MITF is oncogenic [27] indicate that further studies are needed.
